# Light in the box—photobiological examination chamber with light trap ventilation system for studying fungal surface cultures illustrated with *Metarhizium brunneum* and *Beauveria brongniartii*

**DOI:** 10.1186/s40694-023-00159-w

**Published:** 2023-05-29

**Authors:** Pamela Vrabl, Maria Zottele, Lucia Colleselli, Christoph Walter Schinagl, Laura Mayerhofer, Bianka Siewert, Hermann Strasser

**Affiliations:** 1 Institute of Microbiology, University of Innsbruck, Technikerstraße 25, 6020 Innsbruck, Austria; 2 Department of Biotechnology & Food Engineering, MCI-The Entrepreneurial School, Maximilianstraße 2, A-6020 Innsbruck, Austria; 3 Institute of Pharmacy/Pharmacognosy, Center for Molecular Biosciences Innsbruck (CMBI), Center for Chemistry and Biomedicine, University of Innsbruck, Innrain 80-82, A-6020 Innsbruck, Austria

**Keywords:** Entomopathogenic fungi, Fungal photobiology, Light box incubator, Photo-illumination setup, Visible light, LED-technique, Surface culture, Extrolite, Conidia, Morphology

## Abstract

Due to their versatile way of life as saprophytes, endophytes, and entomopathogens, fungi of the genera *Metarhizium* and *Beauveria* are exposed to varying illumination conditions in their natural habitats, which makes a thorough adaptation to light very likely. While the few available studies for these genera support this assumption, research in this field is still in its infancy and the data material restricted to only a few fungal species. Thus, the aim of this work was to explore how light influences growth, conidial production and secondary metabolite formation of two industrial relevant strains of *M. brunneum* (MA 43, formerly *M. anisopliae var. anisopliae* BIPESCO 5/F52) and *B. brongniartii* (BIPESCO 2). To achieve this, we constructed an easily adjustable illumination device for highly standardized photophysiological studies of fungi on Petri dishes, the so-called LIGHT BOX. With the aid of this device, *M. brunneum* and *B. brongniartii* were grown on S4G or S2G agar at 25 °C for 14 days either in complete darkness or under constant illumination with red light (λ_peak_ = 635 nm), green light (λ_peak_ = 519 nm) or blue light (λ_peak_ = 452 nm). In addition, for each wavelength the effect of different illumination intensities was tested, i.e., intensities of red light ranging from 22.1 ± 0.1 to 136.5 ± 0.3 µW cm^-2^, green light from 16.5 ± 0.1 to 96.2 ± 0.1 µW cm^-2^, and blue light from 56.1 ± 0.2 to 188.9 ± 0.6 µW cm^-2^. Both fungi strongly responded in terms of growth, conidial production, pigmentation and morphology to changes in the wavelength and irradiation intensity. The wavelength-dependent production of the well-known secondary metabolite oosporein which is secreted by the genus *Beauveria* in particular, was also increased under green and blue light exposure. The established LIGHT BOX system allows not only to optimize conidial production yields with these biotechnologically relevant fungi, but also allows the photobiological exploration of other fungi.

**Supplementary Information:**

The online version contains supplementary material available at 10.1186/s40694-023-00159-w.

## Background

Light is an important influential factor of the fungal life cycle that has long been known to affect sexual or asexual reproduction, growth, virulence, or secondary metabolism [1–6]. Serving as environmental stress-signal which provides a fungus with information about time or its current location [7, 8], light has received increasing attention as it appears to impact more metabolic levels than initially thought. Indeed, a recent study with *Neurospora crassa* highlighted that up to 31% of all expressed genes in this organism responded to light exposure [9]. This surprisingly high percentage underlines that the role of light in fungi is at best poorly understood [6, 10, 11], especially as only a handful of fungi, mainly *N. crassa* and *Aspergillus nidulans*, have been studied in detail. However, understanding fungal photo responses is far beyond being of mere academic interest. Considering, for example, the abundance of plant–fungi interactions that range from fungal plant pathogens causing severe crop loss worldwide [12, 13] to endophytic fungi which may foster plant growth [14–17], the question of how light influences these organisms and their virulence is of high economic interest.

Fungi of the agriculturally and biotechnologically important genera *Metarhizium* and *Beauveria* exhibit a versatile way of life as (rhizosphere competent) saprophytes, endophytes, or entomopathogens [18–21]. Amongst other factors, these different ecological niches vary in their respective light conditions that range from absolute darkness (e.g. within the soil or the insect), to the green-dominated phyllosphere [3, 8, 22, 23] or to sun (and thus UV-) exposed surfaces. Thus, the assumption suggests itself that these fungi have evolved mechanisms to cope with (fast) changing light conditions including UV-stress. Indeed, although photo responses in *Metarhizium* and *Beauveria* species are scarcely explored, the little available data support a thorough adaptation of these organisms to light. Several studies highlight that in these fungi light induces changes in growth, morphology, virulence, conidiation, germination as well as stress tolerance [24–32].

So far, the majority of light studies with fungi in general, but also with respect to *Metarhizium* and *Beauveria* species in particular, are performed on Petri dishes. This way of cultivation allows fungi to grow on surfaces and provides easy handling as well as the possibility to incubate several experimental series at once. As there is no standardised procedure for photobiological studies with fungi, the literature offers a wide range of methods for illuminating fungi grown on Petri dishes. These methods include colour filters with defined transmission spectra placed on the Petri dish lid [5, 25, 33], modified incubators with specific lamps or custom-made devices (e.g., [2, 4]), and commercial growth cabinets with integrated illumination (e.g., [4, 34]). Only one recent paper describes for the first time a targeted light incubation from below, thus enabling the LED light source to be replaced more easily [35].

Exploring fungal light responses is far from being trivial and demands a high level of experimental standardization. Considering the stress-signal-function of light [7], how Petri dish grown fungi are subjected to different light spectra may already impact the metabolic response and thus the quality of the produced data. For example, the choice of luminant (i.e. bulb versus LED) can make a considerable difference in heat development during the experiment, which can become problematic if not compensated, since some fungi such as *Metarhizium brunneum* are considerably temperature sensitive [36]. Unwanted light impulses even in the range of seconds (e.g., by opening the lid of an incubator system within a room without controlled light conditions) can already alter fungal metabolism [25, 30, 37] and thus distort the observed photo response. While it is evident that the choice of wavelength and the applied intensity play a key role in fungal light responses [11], also the full width at half maximum bandwidth (FWHM) of the illumination conditions is decisive: the light source might comprise significantly different wavelengths from another color range (albeit in low intensities) and may inadvertently trigger additional photoreceptors [8]. Unfortunately, the illumination conditions, which are the prerequisite for a meaningful interpretation and comparison of data, are not defined clearly in many studies [8], thus considerably hampering the progress in the field.

In the present work, an easy-to-use illumination system with a light-trap-equipped ventilation system for surface cultures was developed, which can be placed in climate chambers. This highly standardized irradiation system allows a variable application of the desired range of light intensities and an easy exchange of LEDs of different wavelengths. Using this device, we explored for the first time the photo response of two industrial strains of *M. brunneum* (MA 43, formerly *M. anisopliae var. anisopliae* BIPESCO 5/F52) and *B. brongniartii* (BIPESCO 2) which were grown as Petri dish cultures in dependence of different irradiation scenarios, including variations in wavelengths and illumination intensity. By using this approach, we were able to demonstrate significant changes in morphology, pigmentation, colony diameter, conidial production and metabolite formation for both fungi.

## Methods

### Fungal isolates and cultivation media

All media and detergents were derived from Merck (Darmstadt, Deutschland). *Metarhizium brunneum* (MA 43, formerly *M. anisopliae var. anisopliae* BIPESCO 5/F52) and *Beauveria brongniartii* (strain BIPESCO 2) were cultivated on Sabouraud-4%-Glucose agar media (S4G) and Sabouraud-2%-Glucose agar media (S2G), respectively, at 25 °C for 2 weeks. The conidia were washed off the plates with sterile 0.1% (wt/vol) Tween 80 solution and stored (1 mL per tube) at −80 °C at a final density of 5 × 10^7^ conidia mL^-1^. Before each experiment, a tube was carefully thawed in a 20 °C water bath and shaken for 20 s on a vortex mixer.

### Principle and schematics of the light box system

For the purpose of this work, a custom-made irradiation device, the so-called LIGHT BOX, with an integrated ventilation system was developed together with Gabriel Colleselli (Ceed Electronics e.U., Innsbruck, Austria).

**Fig. 1:**
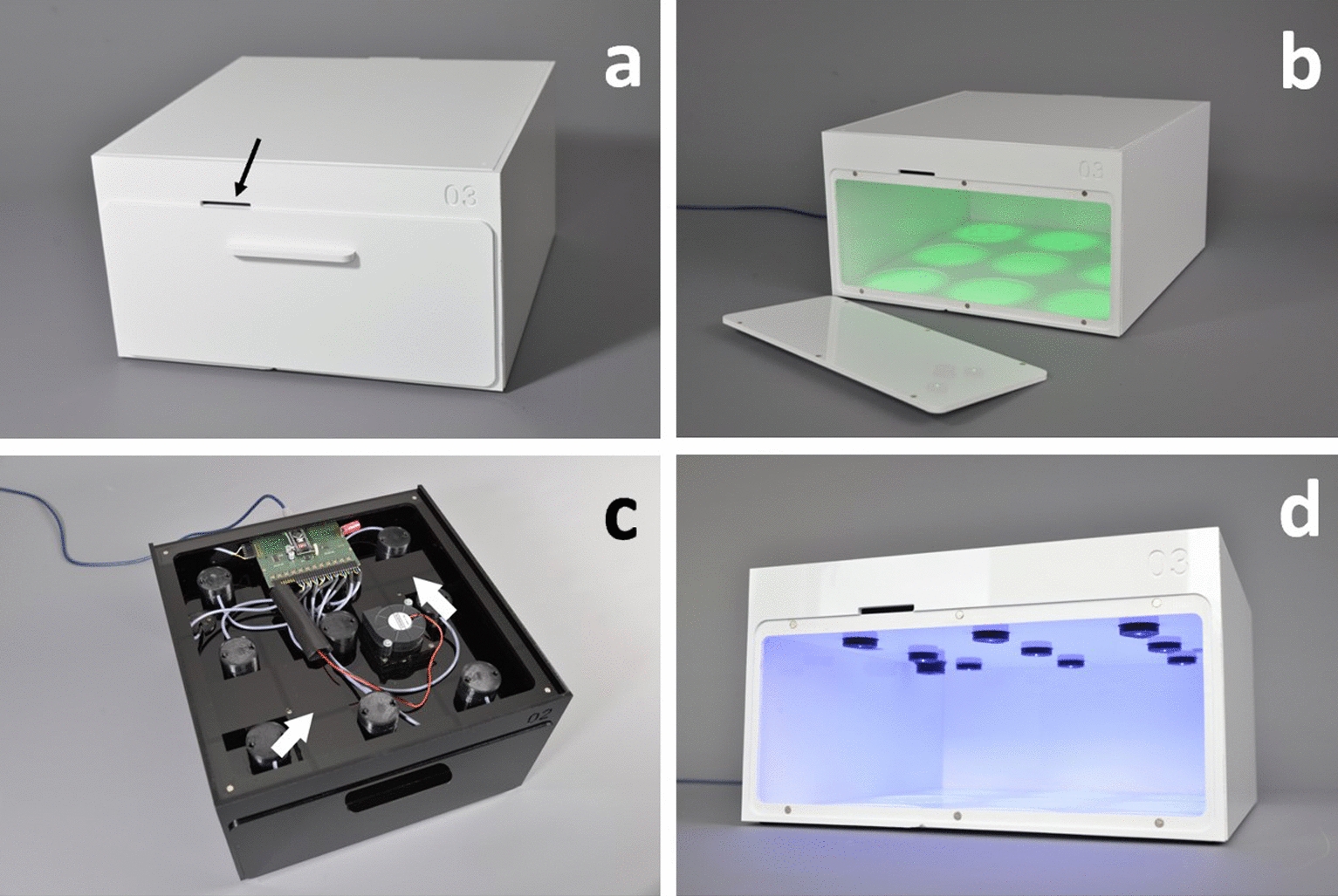
LIGHT BOX made of PMMA. **a** View of the fully assembled LIGHT BOX with closed front-lid and ventilation intake slit (black arrow). **b** View LIGHT BOX with opened front-lid and irradiated areas for Petri dishes on the base plate (illustrated with green light). **c** Top view of the LIGHT BOX control room with magnetic top lid off, showing the electronics, the socked of the LED modules, and the ventilation system with the integrated light traps (arrows). **d** View of the cultivation chambers ceiling with 9 (3 × 3) locked in interchangeable black LED inserts (illustrated with blue light)

**Fig. 2:**
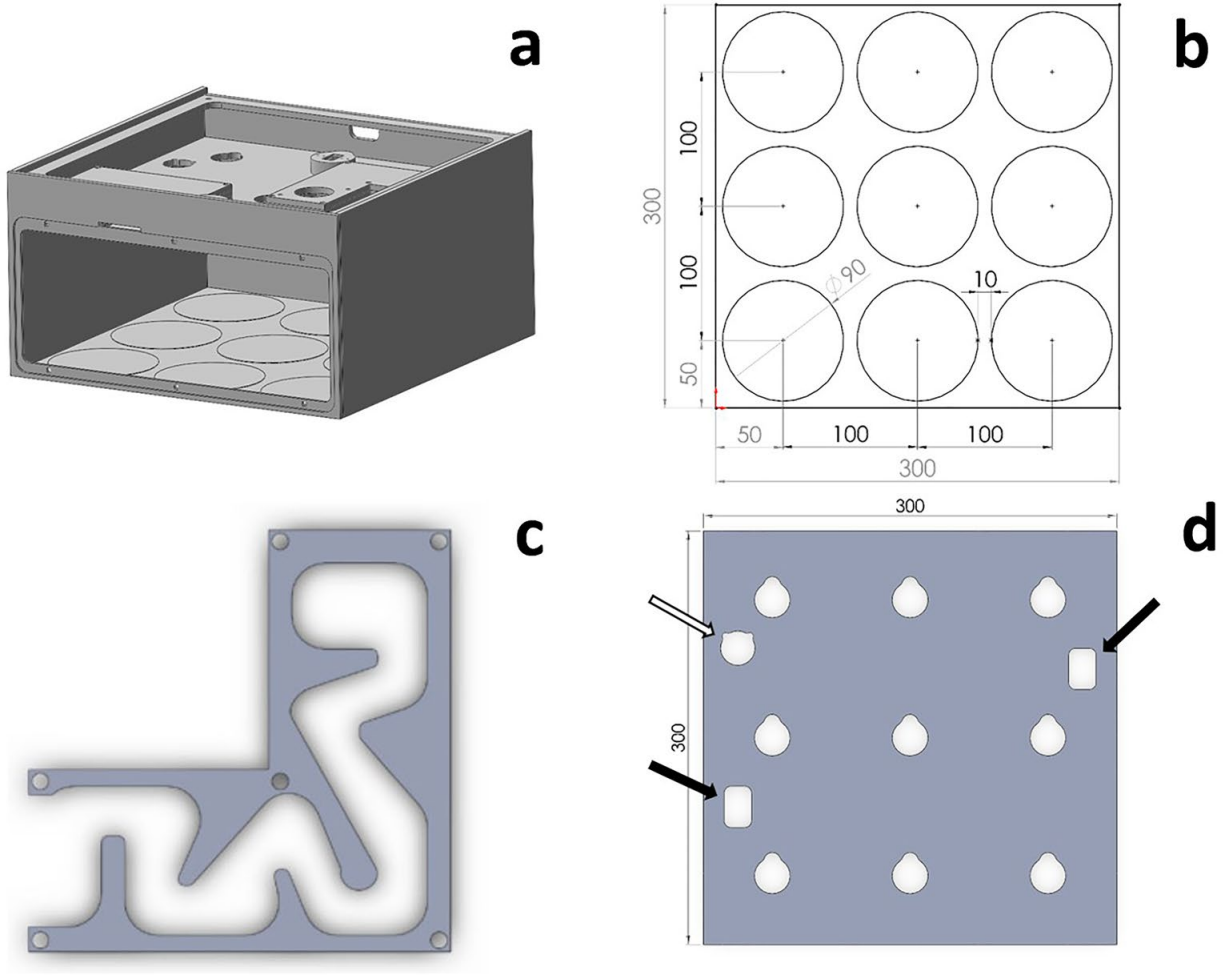
Schematic overview of the LIGHT BOX. **a** Overview basic structure of the housing showing the cultivation chamber and the control room without top cover. **b** Base plate with nine round recesses (diameter 90 mm) milled into the base plate to precisely define the position of the Petri dishes. **c** Meandering duct of the light trap in the ventilation system, which allows the entry of (humid) air without any light contamination. **d** Ceiling of the cultivation chamber with nine cut-outs for the LED inserts with key-lock principle, two further cut-outs for the ventilation system (black arrows), and one cut-out for sensor mounting (white arrow). All dimensions are given in mm

**Fig. 3:**
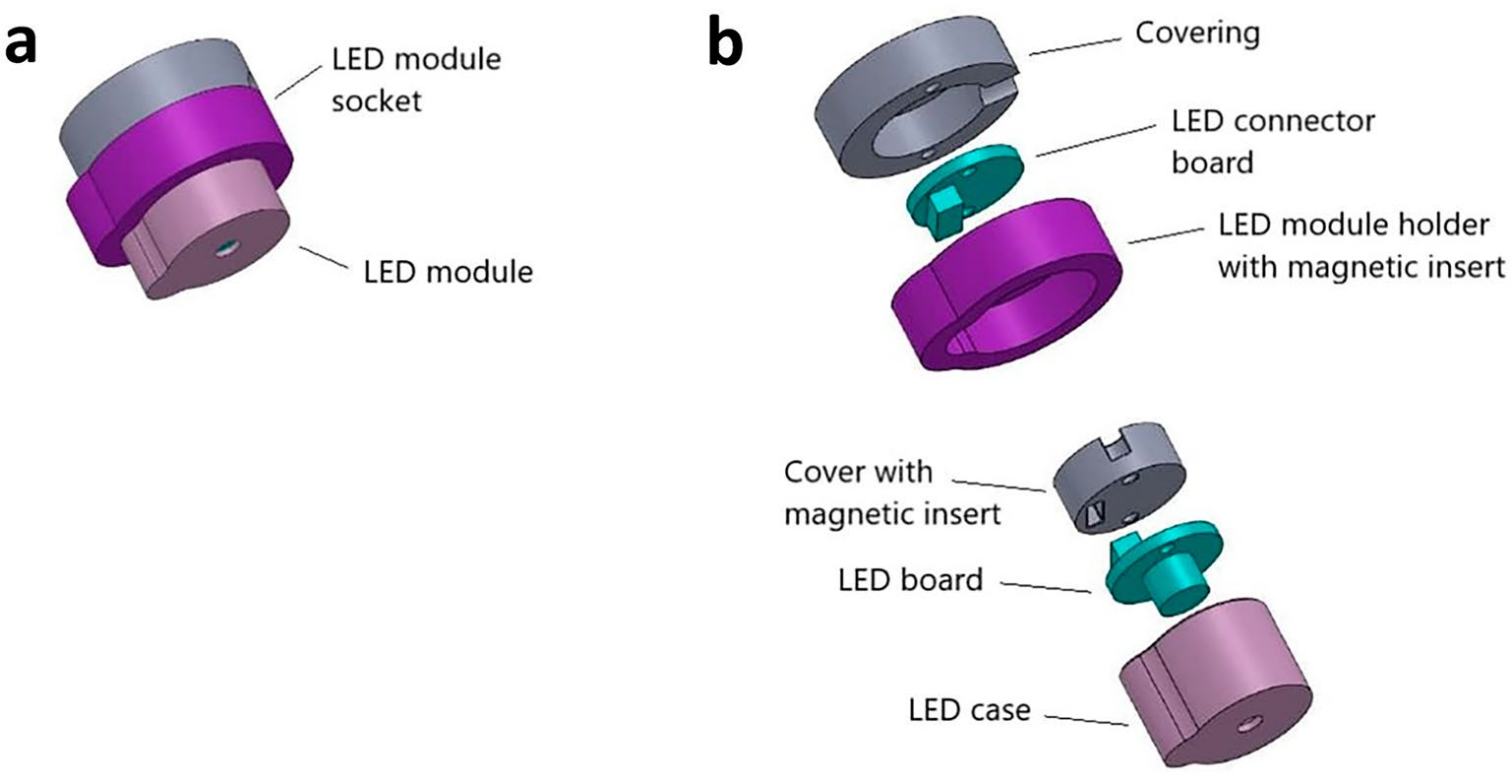
Overview of the LED insert connector system with key-lock principle. **a** Assembled LED connector system consisting out of several modules, which, due to its defined shape, allows only one insertion direction into the socket. **b** Single elements of the LED module in the order of their assembly

Overall, the LIGHT BOX system consisted of several elements (Figs. 1, 2): (i) A cultivation chamber with (ii) a false ceiling into which the LED lamps were inserted via a newly designed plug-in system (Fig. 3). The false ceiling separated the (iii) control room from the cultivation chamber. The control room housed the electronics, the sockets of the LED modules and the light trap of the ventilation system. (iv) Parameters like light intensity, day-night cycle or degree of ventilation were adjusted via a specifically designed software. As material PMMA (poly(methyl methacrylate)) was chosen for the housing due to its advantageous chemical and technical properties. These include but are not limited to its inertness for microorganisms, high hardness and resistance to non-polar solvents, weak acids and alkaline solutions, water, greases and oils [38].

The cultivation chamber is closable via a magnetic front lid (Fig. 1a, b) and offers sites for up to nine Petri-dishes (arranged 3 × 3; see Figs. 1b and 2b). For each single position wells were milled into the base plate for an exact placement of the cultures (sketch base plate see Fig. 2b). The distance between the single Petri-dishes was 10 mm. The nine LED inserts (Fig. 2d) provided an independent and exact illumination for each Petri dish below.

To allow an easy and fast exchange of the LED lamps with different wavelengths, a magnetic LED plug-in system was developed (Fig. 3). The plug-in system of the LED insert consisted of two elements (Fig. 3a), namely the LED socket and the LED module element, which were designed according to a key-lock principle to protect the connectors and to avoid a faulty connection. The LED socket was part of the false ceiling and contained the LED connector board and a magnetic LED module holder with a notch, which were protected by a covering (Fig. 3b). While the LED sockets were fixed elements, the LED modules were interchangeable. Each LED module element comprised the LED-board with the respective SMD LED in the desired wavelength and viewing angles between 110° and 140° (see Additional file 1: Table S1). This LED-board was mounted between a 3D printed LED case with a recess and a cover with a magnetic insert. The irregular-shaped LED-cover allowed only one insertion direction of the LED module into the LED socket. A potential negative overlapping of the LED lighting cones on the cultivation surface was avoided by mounting an adapted aperture in the LED module: The existing light emission opening was adapted until the desired diameter of the irradiated surface was achieved. Irradiation intensity and time interval were adjusted with a specifically designed software (Additional file 1: Fig. S1).

The control room, which was placed above the false ceiling, contained also a specially designed ventilation system that included a light trap (Figs. 1c and 2c). This ventilation system was developed for several reasons: Our goal was to establish a controlled irradiation system, which can be operated in a climate chamber, meaning that the LIGHT BOX would be tempered externally. The ventilation system should provide the LIGHT BOX’s cultivation chamber with the climate chamber’s controlled air humidity. In addition, constant aeration should prevent the cultures from shifting unintentionally into anoxic conditions—a problem that is frequently overlooked in literature. Since the ventilation system is an open system, there was a certain risk of light entering the light chamber. To eliminate unwanted light contamination, the ventilation system was combined with a light trap. The combined ventilation/light-trap system was designed as follows (Fig. 1a, c): Air inlet and air outlet to the cultivation chamber were placed approximately diagonally from each other in the front (inlet) and in the back (outlet), respectively. A fan (Winsinn dual ball bearing fan, 24 V-DC, Shenzhen Weixing Elecronic Technology Co., Ltd., in Shenzhen, Guangdong, China) installed above the air outlet provided a controlled air influx through the cultivation chamber. Both, the air inlet and air outlet contained a right-angled, meandering duct that served as a light trap (Fig. 2c). The air outlet from the cultivation room led into the control room, from where the air escaped through the opening for the USB port. Also, the ventilation intensity was adjusted via the software (Additional file 1: Fig. S1).

### Evaluation and calibration of the light box system

The spectral composition of the LED-lamps (Additional file 1: Table S1 and Fig. S2) was measured with a modular spectrometer (Ocean Optics Maya 2000 Pro, Grating HC-1 200 nm–1050 nm, Optical Fiber 600 µm, Ocean Insight, Orlando, Florida, USA).

The light intensity in the centre of the Petri dish was determined using a radiometer (Thorlabs PM100D, Silicon Power Head 200 nm–1100 nm, Thorlabs, Newton, New Jersey, USA) with various percentual intensity settings of the software (resulting calibration curve see Additional file 1: Fig. S3). In addition, the light distribution across one Petri dish position was measured at the Petri dish centre, half centre, and the border using a 30% intensity level in the software (Additional file 1: Table S2 and Fig. S4). The illumination homogeneity was constant from the centre to half of the Petri dish radius (i.e., less than 6% variability), from where the intensity decreased to about 50% (red and blue light) and 25% (green light) of the value at the centre, depending on the wavelength used (Additional file 1: Table S2). For the Petri dish experiments carried out in this work, this means that most of the mycelium of the *M. brunneum* colonies and all *B. brongniartii* colonies were illuminated under constant conditions (exemplified by two cultures in Additional file 1: Fig. S4).

For each applied wavelength, the resulting illumination intensity was determined in µW cm^−2^ for the software settings 0%, 5%, 10%, 20%, 30%, and 40% (calibration see Additional file 1: Fig. S3). In all experiments, the maximum illumination intensity in the LIGHT BOX was limited to 30%, since beyond the 40% energy output, especially with the blue light LEDs, an increased temperature development in the LIGHT BOX could be determined. Despite the ventilation system, this temperature increase was only compensated to a limited extent.

### Cultivation and irradiation conditions

Light exposure experiments were conducted at 25 °C employing the LIGHT BOX either in darkness or irradiated with λ_peak_ = 452 ± 18.7 nm (blue), 519 ± 38.2 nm (green) or 635 ± 18 nm (red) with different intensities ranging from 5–30% in the software setting (for corresponding irradiation intensities see Additional file 1: Figure S3, Tables S3 and S4).

S4G and S2G agar plates, containing 20 mL agar media each, were inoculated with an inoculation needle in the centre of each Petri dish with *M. brunneum* and *B. brongniartii*, respectively, and incubated for 2 weeks. As control, conidia were incubated in the same boxes without any irradiation. Four replicates were performed for each condition. Since in total two structurally identical boxes were used for this study, it was possible to perform two conditions in parallel.

### Evaluation of conidial production and growth

Fungal growth under the different applied conditions was assessed by measuring the diameter of the colonies after 2 weeks. Conidial production was evaluated by taking agar plugs with a cork borer (9 mm inner diameter). Plugs were taken in between the centre and the border of the colony. The core samples were placed in a tube containing 1 mL sterile Tween 80 solution (0.1%), shaken for 20 s on a vortex mixer, and sonicated for 30 s before counting conidia in a Hemocytometer (Thoma chamber). Conidial concentrations were calculated as conidia per square cm. The germination ability of the conidia was estimated by plating the suspension onto agar media plates, which were then incubated at 25 °C for 2 weeks followed by an assessment of the colony forming units (CFU).

### Reagents and materials for mycochemistry

All chemicals and reagents were sourced from VWR (Vienna, Austria) or stated otherwise. Solvents used for the extraction were purified by distillation. Solvents used for the HPLC analysis were purchased in HPLC grade. Ultrapure water was prepared with the Sartorius arium^®^611 UV purification system (Sartorius AG, Göttingen, Germany). Boric acid was sourced from Merck KGaA (Darmstadt, Deutschland). Freeze-drying was done with the SP VirTis BenchTop Pro with Omnitronics from SP (Stone Ridge, NY, US). A coffee grounder was used for the initial grinding. The Sartorius Cubis^®^-series balance (Sartorius AG, Göttingen, Germany) and Mettler Toledo AB54 (Mettler-Toledo GmbH, Vienna, Austria) were utilized as well as the ultrasonic bath Sonorex RK52 from Bandelin (Berlin, Germany). Centrifugation was done with the Function Line Centrifuge from VWR (Vienna, Austria). Pipettes and pipette tips from STARLAB International GmbH (Hamburg, Germany) and Eppendorf AG (Hamburg, Germany) were utilized. Filtration prior HPLC analysis was performed with Chromafil^®^Xtra PTFE-45/13 filters from Macherey-Nagel (Düren, Germany). A Shimadzu HPLC/UPLC-UFLC XR instrument and a Synergi 4u Hydro-RP 80A 150x4.6 mm were utilized. The Britton-Robinson buffer with a pH of 5.5 was prepared from: acetic acid (5.13 mL, 1.65 M), phosphoric acid (8.4 mL, 1.02 M), sodium hydroxide (6.84 mL, 2.5 M), and boric acid (53.1 mL, 0.16 M). After mixing, the solution was filled up to a total volume of 300 mL utilizing distilled water. The pH of 5.5 was set by adding phosphoric acid after diluting the buffer with methanol to a ratio of 3:7.

### Extract preparation

The solid medium containing one colony was cut into nine pieces and transferred into a beaker. After freezing at −80 °C, the material was lyophilized for 24 h and ground. After homogenization, 2 samples, each 200 g were drawn. The extraction was performed by ultrasonication (10 min) employing the BR5.5-MeOH buffer as solvent (5 mL). The supernatant was decanted after ultracentrifugation, and the extraction step was repeated twice. The combined supernatants were dried under a stream of air and under light protection. A part of the concentrated extracts (5 g) was resolved in the BR5.5-MeOH buffer, filtered, and submitted to an HPLC-DAD measurement.

### HPLC analysis

The following settings resulted in satisfying outcomes: T_oven_ = 40 °C, flow 1.4 mL min^−1^, injection volume of 5 µL. As mobile phase A, ultrapure water supplemented with 0.1% acetic acid and 0.9% formic acid was used, as mobile phase B served acetonitrile with the same supplements. The gradient was set to 20% B at t = 0 min and reached 50% B after 14.99 min. Via a calibration curve of oosporein (r² = 0.9949), the LOD and LOQ were calculated [39] to be 0.209 and 0.666 mg mL^-1^, respectively. The chromatograms were analyzed with Origin 2020 (OriginLab Corporation, Northampton, MA, USA). The integration results of peak 1 (unidentified mixture of approx. three compounds) and peak 2 (oosporein) were normalized by the extract yields, and then plotted with R studio (Version 1.3.1093).

### Statistical analysis

The effect of different irradiation regimes on the radial growth and conidial production of the two different fungi was assessed with a two-way ANOVA (interaction) and Tukey-HSD post hoc test. Analyses were carried out with R studio version 1.3.959 graphs were plotted with Origin 2020 (OriginLab Corporation, MA, USA).

## Results

In this study, a user-friendly and easily adjustable illumination system, hereafter referred to as LIGHT BOX (Figs. 1, 2, 3), is proposed to meet the urgent need for highly standardised equipment for photophysiological studies of microorganisms cultured on solidified media.

### Effect of light on growth, conidial production, and morphology of *M. brunneum and B. brongniartii*

**Fig. 4:**
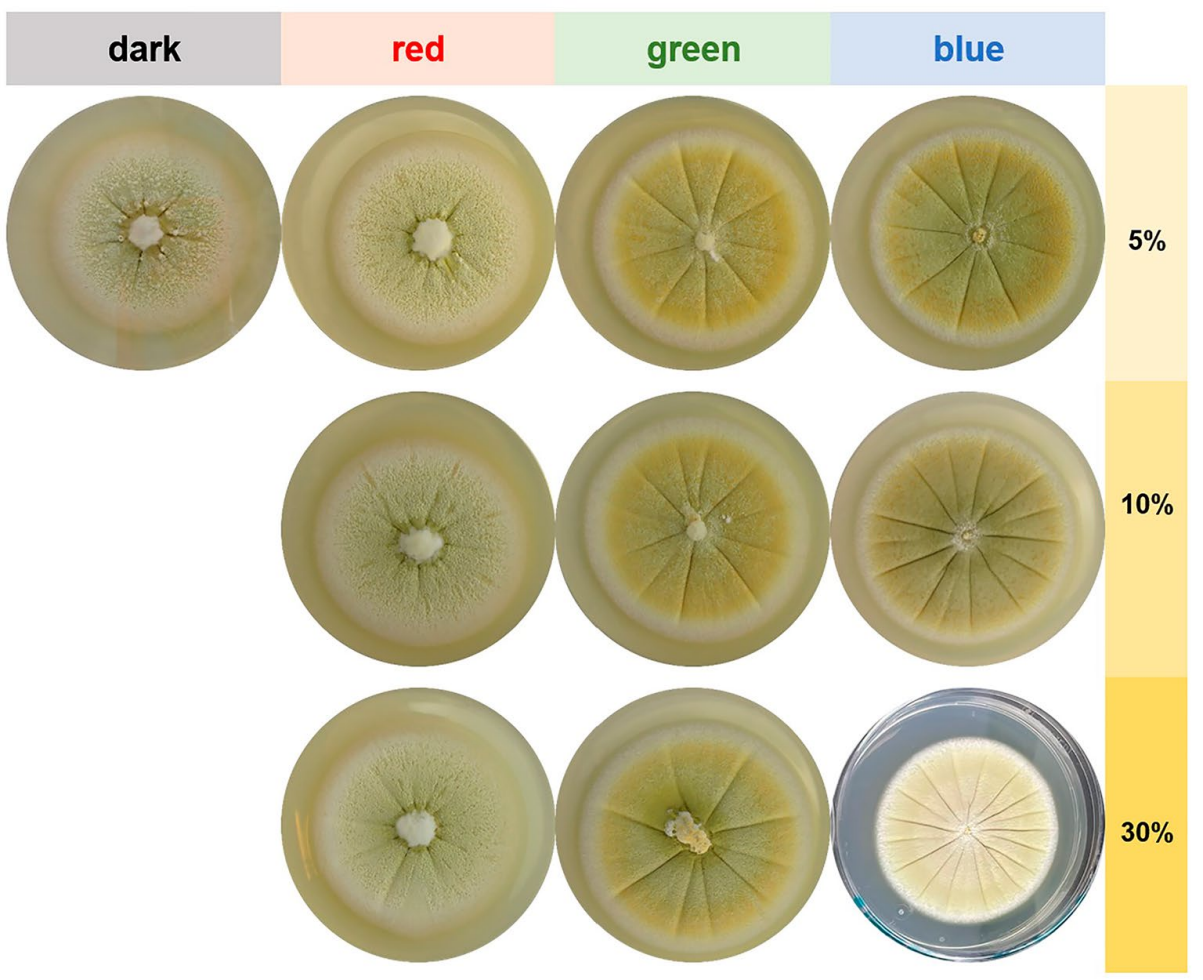
Colony appearance of *M. brunneum* grown on S4G medium in dependence of different irradiation regimes. Cultures were cultivated for 2 weeks either in complete darkness or exposed to blue light (λ_peak_ = 452 nm), green light (λ_peak_ = 519 nm) or red light (λ_peak_ = 635 nm). Corresponding illumination intensities in µW cm^-2^ are given in Additional file 1: Table S3. Per condition, a typical colony is shown

**Fig. 5:**
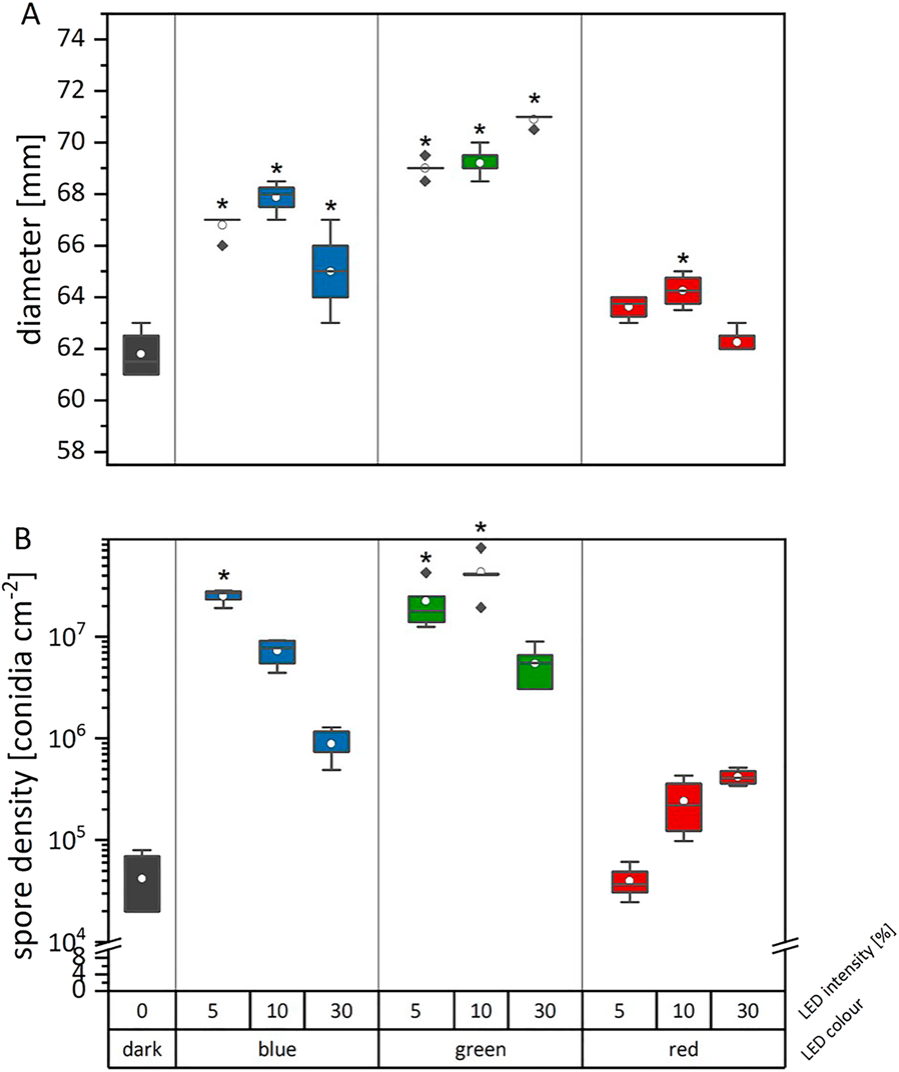
Radial growth (culture diameter, **A**) and conidial density (**B**) of *M. brunneum* grown on S4G medium in dependence of different irradiation regimes. Cultures were grown for 2 weeks either in complete darkness or exposed to blue light (λ_peak_ = 452 nm), green light (λ_peak _= 519 nm) or red light (λ_peak_ = 635 nm). The asterisk indicates the significant difference between treatment and dark control. Corresponding illumination intensities in µW cm^-2^ and raw data are given in Additional file 1: Table S3. Data are means of at least quadruplicate cultures Per condition

**Fig. 6:**
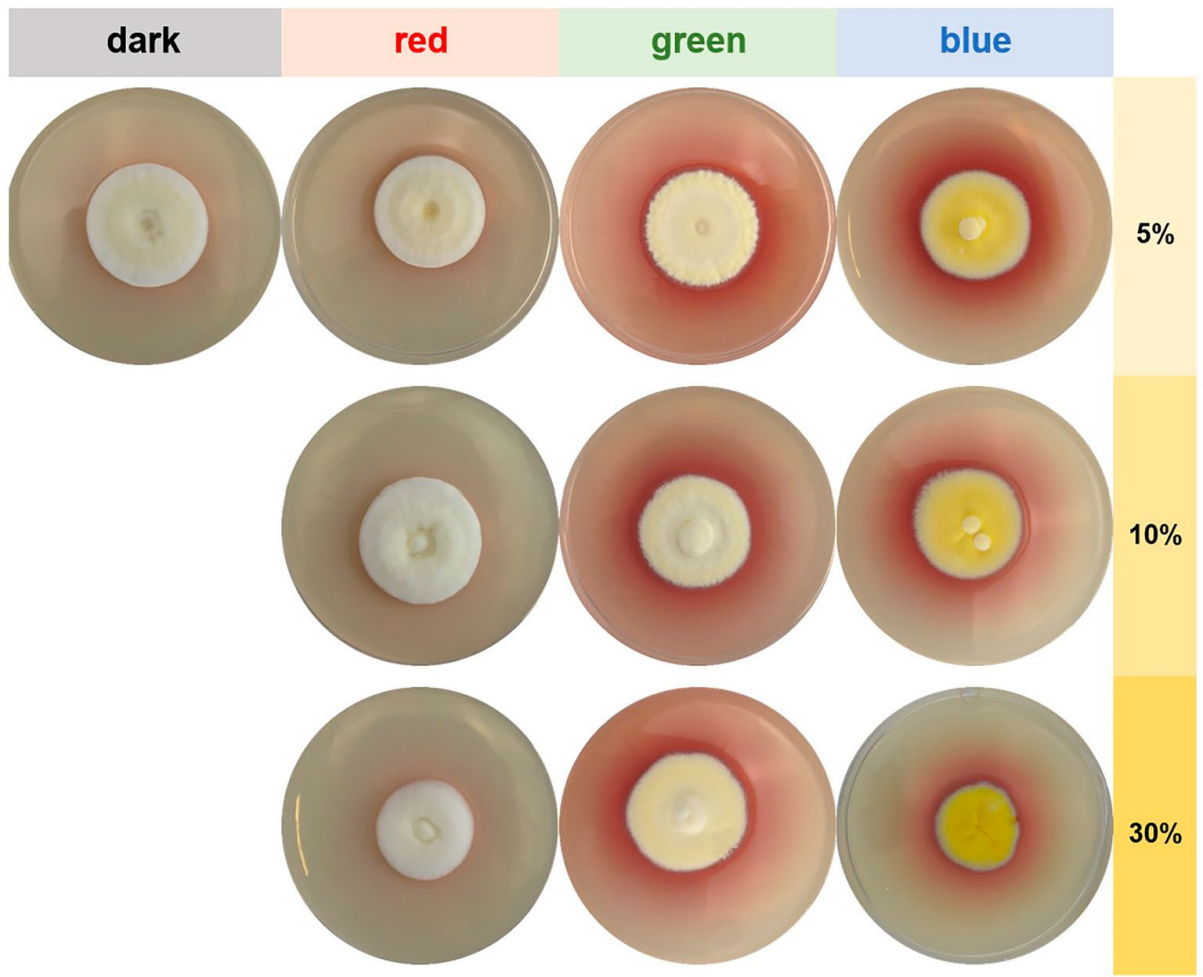
Colony appearance of *B. brongniartii* grown on S2G medium in dependence of different irradiation regimes. Cultures were cultivated for 2 weeks either in complete darkness or exposed to blue light (λ_peak_= 452 nm), green light (λ_peak _= 519 nm) or red light (λ_peak _= 635 nm). Corresponding illumination intensities in µW cm^-2^ are given in Additional file 1: Table S4. Per condition, a typical colony is shown

**Fig. 7:**
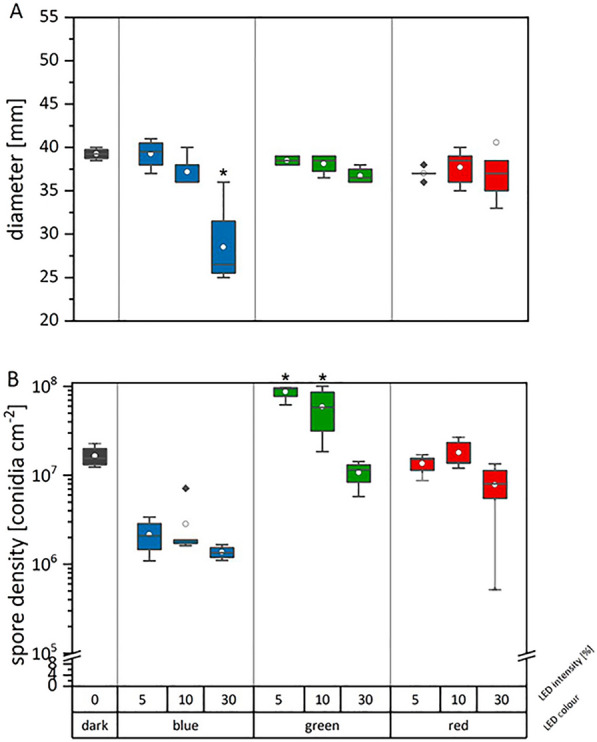
Radial growth (culture diameter, **A**) and conidial density (**B**) of *B. brongniartii* grown on S2G media in dependence of different irradiation regimes. Cultures were grown for two weeks either in complete darkness or exposed to blue light (λ_peak_ = 452 nm), green light (λ_peak_ = 519 nm) or red light (λ_peak_ = 635 nm). The asterisk indicates the significant difference between treatment and dark control. Corresponding illumination intensities in µW cm^-2^ and raw data are given in Additional file 1: Table S4. Data are means of at least quadruplicate cultures Per condition

Incubation with the different wavelengths of visible light had a significant influence on growth and morphology of *M. brunneum* and *B. brongniartii* (Figs. 4, 5, 6,7). Especially cultures irradiated with green and blue light showed differences in colony morphology compared to the controls, i.e., cultures grown in darkness.

#### *Metarhizium brunneum*

Independent of the irradiation regime, *M. brunneum* produced round, filamentous colonies (Fig. 4). The colonies grew umbonate, whereby the size of the middle elevation decreased with decreasing wavelength (highest elevation in darkness or red-light irradiation, lowest elevation after blue light irradiation). Blue- and green-light incubated colonies had a yellow greenish color, whereas colonies grown under red light or in darkness were very light with a slight hint of light-green. The surface changed with the blue and green light exposure from rough to smooth-powdery.

*M. brunneum* cultures grown in the dark had the lowest radial growth, i.e., 61.8 ± 0.9 mm in diameter, after 2 weeks of incubation (Fig. 5, Additional file 1: Table S3). All further irradiation regimes, except for red light irradiation (5% and 30% software setting, equaling an irradiation of 22.1 ± 0.1 µW cm^−2^ and 136.5 ± 0.3 µW cm^−2^ for 14 days), showed colonies significantly larger in diameter compared to the cultures grown in the dark (p < 0.002). Over the whole experimental series, the largest colony diameters, i.e., 70.9 ± 0.2 mm, were found for cultures incubated with green light for 14 days at 96.2 ± 0.1 µW cm^−2^ (equaling 30% gain setting in the software). The conidial production of *M. brunneum* was also influenced by the light conditions (Fig. 5, Additional file 1: Table S3). Overall, exposure to blue and green light led to a significantly higher conidial yield per area compared to dark incubated cultures (p < 0.04). Those differences were caused by higher yields after incubation with 5% intensity blue light or 5–10% intensity green light (p < 0.006). Blue light incubation with stronger intensities reduced the conidial yield. However, the yield of blue light exposed cultures was still 21–175 times higher than of those grown in darkness.

#### *Beauveria brongniartii*

Independent of the irradiation regime, *B. brongniartii* produced round, filamentous colonies (Fig. 6). Typical for dark and red-light incubated plates were fluffy, elevated colonies with a crateriform centre. The color of these colonies was white with a creamy color in the middle. In contrast, green- and blue-light exposed colonies grew flat with a raised, fluffy centre. Green incubated colonies had a homogenous creamy color, whereas blue incubated colonies showed a yellow color. Under these conditions also the production of a reddish-purple pigment, usually attributed to oosporein, was considerably enhanced. To confirm this observation, a targeted HPLC analysis was performed with this whole experimental series (see section" Targeted metabolite analysis of B. brongniartii cultures"). The radial growth of *B. brongniartii* was only influenced by blue light irradiation at the 30% software setting, which corresponds to a constant illumination of 188.9 ± 0.6 µW cm^-2^ for 14 days (Fig. 7, Additional file 1: Table S4). The diameter of the colonies grown with this irradiation regime (i.e., blue light 30%) was significantly lower (p < 0.001). In addition, the conidia density was negatively influenced by increasing the intensity of green light (r =−0.83, p < 0.001) (Additional file 1: Table S5). Significantly more conidia (p < 0.002) could be harvested from green-light irradiated plates at 5% and 10% software setting (equaling 16.5 ± 0.1 µW cm^-2^ and 23.1 ± 0.0 µW cm^-2^, respectively).

### Targeted metabolite analysis of *B. brongniartii* cultures

**Fig. 8:**
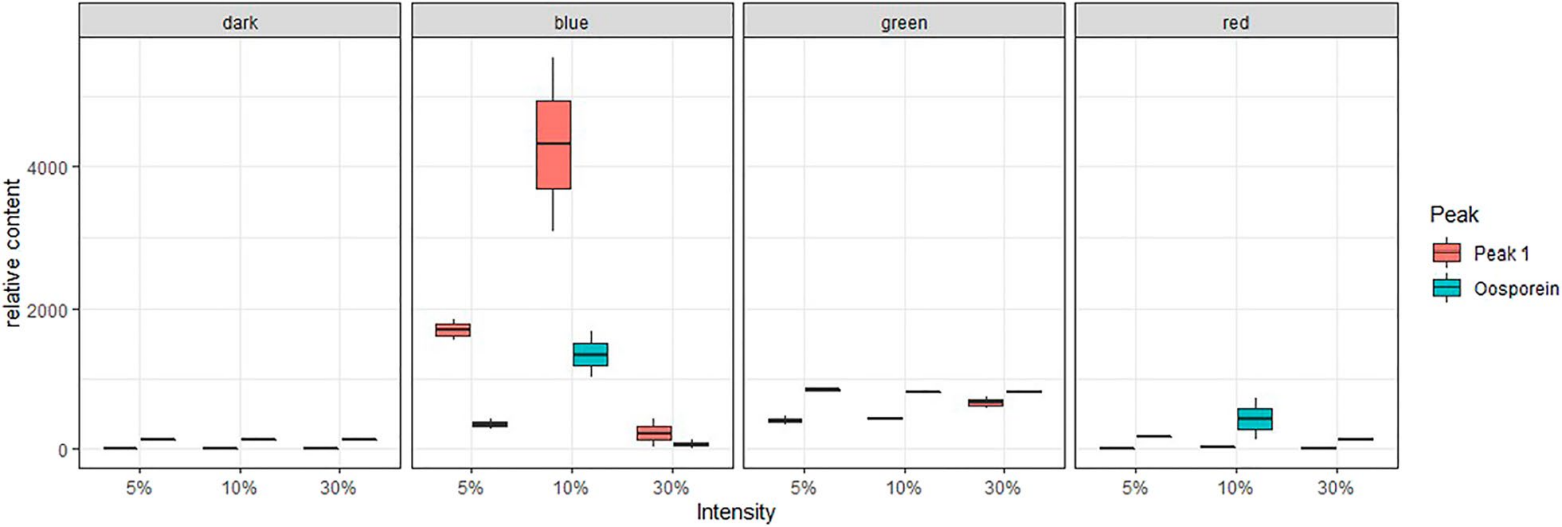
Relative content of oosporein and “Peak 1” detected in the cultures of *B. brongniartii* in respect to the utilized irradiation source and power

Corresponding to the different light frequencies, we found a light-dependent accumulation of a reddish pigment in the medium (Fig. 6). Since the reddish coloration was increased under green and blue light exposure, a targeted metabolite analysis was performed for the whole experimental series based on previous analytical work [40, 41]. Two major peaks were observed in the HPLC chromatogram (Additional file 1: Figure S5 and S6), of which one could be attributed to oosporein. The other, yet unknown ‘Peak 1’ is possibly a mixture of yellowish pigments found under lower irradiation wavelengths in this organism (Fig. 6). Both metabolites showed a clear light dependency and were produced in higher amounts under green and blue light irradiation (Fig. 8). These data are in line with the photo-material gained for this experimental series (Fig. 6).

## Discussion

This study proposes a user-friendly and easily adjustable illumination device, that meets the urgent need for highly standardized equipment for photophysiological studies of microorganisms cultivated on solidified media. One of the main problems we aimed to solve with this photo illumination set-up was that most commercially available incubators with controlled illumination do not offer controlled air humidity conditions like climate chambers. Air humidity, however, is known to strongly influence microbial growth and physiology [42]. Thus, the primary premise in this work was to establish an illumination system with a light-tight air ventilation system that can be placed in existing climate chambers as independent units. The illumination device thereby offers the experimenter the possibility to benefit from the controlled air humidity conditions of the climate chamber, if the experimental needs demands it.

Apart from the central premise of a ventilation system, the developed illumination device had to meet further demands: Firstly, we used materials that did not negatively interfere with the cultivation, e.g., by chemical evaporation of the material itself or adhesives. The chosen material, PMMA, is used in a variety of medical and healthcare applications and proved resistant to common disinfectants so that it meets the necessity of cleaning and disinfection in the microbiologist’s everyday laboratory life [38]. Secondly, the illumination was designed in such a way that it is homogenous and easily adjustable in terms of wavelength, intensity, or day-night-rhythm. Thirdly, the designed cultivation chamber is completely light-tight so that the cultures are not influenced by even random light contamination. Finally, the illumination system was equipped with a series of sensors to follow the progress of temperature and air humidity conditions during the incubation time. Thus, potential interferences such as a short-time failure of the surrounding climate chamber can be detected.

With the help of the new LIGHT BOX system we were able to carry out a large number of photobiological/physiological investigations with the two well characterized mitosporic ascomycetes *M. brunneum* (MA 43, BIPESCO 5) and *B. brongniartii* (BIPESCO 2) under standardised conditions, as is generally required by good microbiological laboratory practice [43]. In the process, we were able to gain initial important insights into our two production strains, which are widely applied as pest control agents in OECD countries [44].

### Influence of light on growth, conidia production, and metabolite production in *Metarhizium brunneum* and *Beauveria brongniartii*

#### Illumination intensities – an often overlooked issue

Light intensity has long been known to have a decisive influence on the light response of fungi [37]. There is a considerable range of illumination intensities that are used for photobiological studies of fungal Petri dish cultures (Table 1). The bandwidth comprises studies using extremely low exposure intensities (< 1 µW cm^-2^), low (1–100 µW cm^-2^) and medium exposure intensities (100–1000 µW cm^-2^), up to high exposure intensities (> 1000 µW cm^-^²). The latter are mostly, but not exclusively, found in studies exploring the effect of high light dosages on fungal biology.

To our knowledge, light studies for *Metarhizium* and *Beauveria* species are typically performed with medium exposure intensities or above (Table 1). With the aid of the LIGHT BOX, we were able to demonstrate that *M. brunneum* and *B. brongniartii* are showing even distinct photo-responses to intensity changes in the low-intensity exposure range. Besides being of ecological interest, this fact has to be taken into account when comparing the data of this study with other studies focusing on *Metarhizium* and *Beauveria* species, which used higher intensity values.

**Table 1:** Examples of applied light intensities on fungal surface cultures

Irradiation intensity range (µW cm^-2^)	Organism	Study
0.009	*Aspergillus nidulans*	[45]
0.2–2	*Mucor circinelloides*	[46]
17–189	*Beauveria brongniartii, Metarhizium brunneum *	Present work
51–90	*Aspergillus nidulans*	[47]
72–145	*Alternaria alternata*	[48]
200–2760	*Metarhizium robertsii*	[49]
220–498	*Metarhizium robertsii*	[4]
280–498	*Metarhizium robertsii*	[29]
380	*Metarhizium robertsii*	[50]
500	*Fusarium solani*	[51]
530–6000	*Metarhizium acridum*	[30]
540	*Metarhizium robertsii*	[52]
800–1200	*Penicillium ochrochloron*	[53]
1100	*Aspergillus nidulans*	[54]
3800	*Alternaria alternata, Aspergillus carbonarius, A. steyrii, A. parasiticus, Fusarium graminearum, Penicillium nordicum, P. verrudosum, P. expansium*	[55]

#### *Metarhizium brunneum*

In agreement with recent studies on *M. robertsii*, *M. acridum*, *M. anisopliae*, and *M. flavoviride* [29–32], we found an intensity-dependent positive effect of blue light on conidial production of *M. brunneum* (Figs. 4, 5). However, while Oliveira *et al*. 2018 [29] did not observe any reaction of *M. robertsii* to green light, we observed that green light exposure influenced *M. brunneum* on several levels: Firstly, the colony coloration and morphology changed when the cultures were exposed to green and blue light. This was further substantiated in a preliminary screening experiment where, with the onset of green light and shorter wavelengths combined with storage of the plates at 4 °C after the end of the experiment, we were able to detect the production of a yet unknown extracellular green pigment. This pigment is a potential photosensitizer as the extract containing this pigment produces singlet oxygen under light exposure [56]. Secondly, similar to blue light, the conidial densities of *M. brunneum* were considerably higher compared to cultures exposed to red-light or grown in complete darkness. Lastly, the largest colony diameters were formed under green-light exposure.

These contrasting results, i.e., no green light effect in *M. robertsii* [29] versus green light effect in *M. brunneum* (present study), might be rooted in the use of different growth media as the nutritional status can be decisive for the photo response [4, 37, 53]. However, since the whole genus *Metarhizium* seems to be devoid of opsins [8], the observed, distinct green light responses are somewhat puzzling. One possible explanation may lie in the nature of the applied green-light source, which typically also emits (albeit in very low intensities) light below 500 nm and thus might have triggered a response of the WC-1 receptor inadvertently [8]. Of interest in this context is that in *Mucor circinelloides* one of the WC-1 orthologs was reported to be involved in green light induced phototropism [46]. Whether this green light sensing via a WC-1 ortholog also applies to *Metarhizium* species remains to be determined.

#### *Beauveria brongniartii*

To our knowledge, the effect of light on the genus *Beauveria* has so far only been explored in the species *B. bassiana*. All available studies on this subject highlight that this species is very light-responsive in terms of morphology, conidial production, stress tolerance, and metabolite production [25–28, 57].

Similarly, we could also demonstrate that *B. brongniartii* is highly light-responsive in terms of culture morphology, conidial production and metabolite excretion (Figs. 6, 7). Interestingly, the conidial production was significantly decreased in cultures exposed to blue light, independent of the applied intensity. Studies exploring the effect of defined wavelengths on *Beauveria* conidiation are rare and difficult to compare due to the vast differences in the methodological approaches. Tong *et al.* 2018 found that for *B. bassiana* the amount of conidia after 7–8 days of incubation was higher in the darkness than under blue light exposure, which is in line with the data of our study. However, Zhang *et al*. 2009 [25] reported the opposite and observed that blue light considerably stimulated the conidial production in *B. bassiana* after 15 days of incubation. Like the controversies found in literature for *M. brunneum*, these contrasting results might be the consequence of nutrient dependent light responses in these fungi.

In this context, the wavelength-dependent production of the red dibenzoquinone oosporein is of high interest. In *Beauveria* species, this extrolite plays a key role during the infection process and is furthermore known for its multitude of biological activities, including antibiotic, antifungal, antiviral, antioxidant or cytotoxic effects [51, 58, 59]. As far as we are aware, a photo-induction of oosporein was only reported once in *B. bassiana* [57]. Our data clearly verify this observation and identify green and blue light exposure as strong enhancer of oosporein production (Fig. 6, 8), thus adding these wavelengths to the few known trigger factors of this secondary metabolite [51].

## Conclusions

Entomopathogenic fungi such as *Metarhizium* and *Beauveria* produce tiny hydrophobic airborne conidia, which constitute the main dispersal, survival and infection unit. These airborne conidia are produced under natural environmental conditions on the surface of infected cadavers. The conidial products are usually produced commercially on solid substrates (e.g., rice, barley), i.e. surface cultures, and one industry effort is to increase yields by manipulating culture conditions, such as starter broth cultures, selection of low-cost substrates and incubation conditions. With the established LIGHT BOX in this work, we are now in the position to investigate a previously underestimated important parameter, namely the influence of different illumination intensities in the visible light range, under standardized incubation conditions. The data gained this study indicate that with comparable little effort the conidial yield of the production strains *M. brunneum* (MA 43, BIPESCO 5) and *B. brongniartii* (BIPESCO 2) can be increased and the production time reduced by 10–20%. In the future, our LIGHT BOX will also be used to explore new indigenous entomopathogenic fungi as well as other biotechnologically and medically relevant fungi. It is expected that several new production protocols and recipes will be developed, and numerous relevant further experiments can be conducted. Basic research will be continued with special emphasis on metabolomics. The aim is to create metabolic profiles, but above all to identify fungal metabolites that are released at different light spectra.

## Supplementary Information

**Additional file 1:** **Figure S1**: User interface of the operation software with options to control the light intensity, the irradiation interval and the ventilation intensity. Beside an overview of the current settings also the current air humidity and the temperature of the cultivation chamber is provided. **Table S1**: Relevant illumination properties of the used light sources. The LED wave length and the respective FWHM were recorded as described in the section Materials and Method. **Figure S2**: Spectral distribution of the different irradiation scenarios used in this work. For better comparison, intensities were normalized. **Figure S3**: Calibration curve software setting of the light box versus measured intensity. Data are means of triplicate measurements. **Figure S4**: Measuring points within one Petri-dish position to evaluate the light distribution within a Petri-dish. center, half radius, border measuring point. Typical *M.n brunneum* and *B. brongniartii *cultures grown at 25 °C for 2 weeks. **Table S2**: Light distribution measured at the center, the half-radius and the border of a Petri dish. The illumination was set to 30% for all tested colors. Data are means of triplicate measurements. **Table S3**: Radial growth and conidia production of *M. brunneum* after 2-week incubation on S4G at 25 °C under different light regimes. Data are means of triplicates. **Table S4**:Radial growth and conidia production of *B. brongniartii* after 2-week incubation on S2G at 25 °C under different light regimes. Data are means of triplicates. **Table S5**: Pearson correlation coefficients for the relation of diameter and conidia per cm^-2^ with the intensity of different light regimes. **Figure S5**: Representative HPLC-DAD chromatograms of the targeted oosporein analysis of *B. brongniartii*. Stationary phase:  Phenomenex Synergi 4u Hydro-RP80A 150 × 4.6 mm. Mobile phase: Water and ACN supplemented with 0.1% acidic acid and 0.9% formic acid. **Figure S6**: HPLC-DAD chromatograms of the analyzed *B. brongniartii *extract grown under blue light. A blue light-absorbing peak was detected at 2.77 min. The peak shape indicates a mixture of different compounds or isomeric forms. Stationary phase:  Phenomenex Synergi 4u Hydro-RP 80A 150 × 4.6mm. Mobile phase: Water and ACN supplemented with 0.1% acidic acidand 0.9% formic acid.

## Data Availability

All data generated or analysed during this study are included in this published article and its supplementary information files.
