# Supplementary material for: Light in the box—photobiological examination chamber with light trap ventilation system for studying fungal surface cultures illustrated with Metarhizium brunneum and Beauveria brongniartii
Source: Fungal Biol Biotechnol. 2023 May 29;10:11. doi: 10.1186/s40694-023-00159-w (PMC10228068; doi:10.1186/s40694-023-00159-w)
Supplement: Supplementary file 1 — Additional file 1: Figure S1: User interface of the operation software with options to control the light intensity, the irradiation interval and the ventilation intensity. Beside an overview of the current settings also the current air humidity and the temperature of the cultivation chamber is provided. Table S1: Relevant illumination properties of the used light sources. The LED wave length and the respective FWHM were recorded as described in the section Materials and Method. Figure S2: Spectral distribution of the different irradiation scenarios used in this work. For better comparison, intensities were normalized. Figure S3: Calibration curve software setting of the light box versus measured intensity. Data are means of triplicate measurements. Figure S4: Measuring points within one Petri-dish position to evaluate the light distribution within a Petri-dish. center, half radius, border measuring point. Typical M.n brunneum and B. brongniartii cultures grown at 25 °C for 2 weeks. Table S2: Light distribution measured at the center, the half-radius and the border of a Petri dish. The illumination was set to 30% for all tested colors. Data are means of triplicate measurements. Table S3: Radial growth and conidia production of M. brunneum after 2-week incubation on S4G at 25 °C under different light regimes. Data are means of triplicates. Table S4:Radial growth and conidia production of B. brongniartii after 2-week incubation on S2G at 25 °C under different light regimes. Data are means of triplicates. Table S5: Pearson correlation coefficients for the relation of diameter and conidia per cm-2 with the intensity of different light regimes. Figure S5: Representative HPLC-DAD chromatograms of the targeted oosporein analysis of B. brongniartii. Stationary phase: Phenomenex Synergi 4u Hydro-RP80A 150 × 4.6 mm. Mobile phase: Water and ACN supplemented with 0.1% acidic acid and 0.9% formic acid. Figure S6: HPLC-DAD chromatograms of the analyzed B. brongniartii extract grow [file 40694_2023_159_MOESM1_ESM.docx]

# Supplementary Files to the Manuscript

**Light in the box - photobiological examination chamber with light trap ventilation system for studying fungal surface cultures illustrated with *Metarhizium brunneum* and *Beauveria brongniartii***

Pamela Vrabl, Maria Zottele, Lucia Colleselli, Christoph Walter Schinagl, Laura Mayerhofer, Bianka Siewert, Hermann Strasser


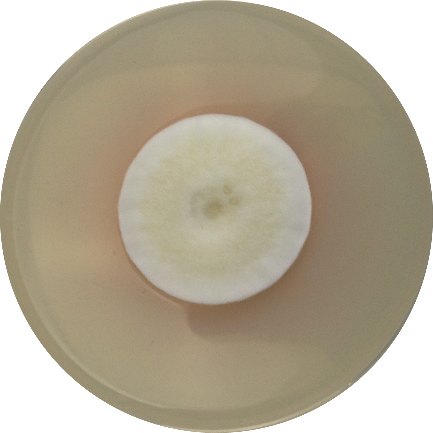

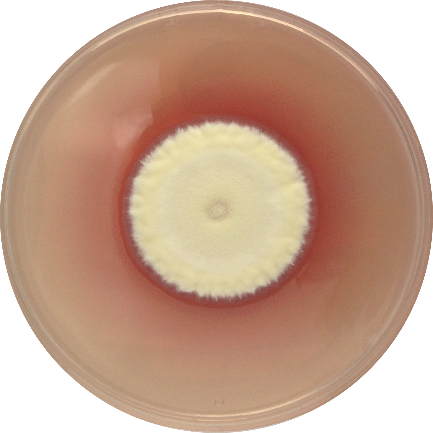

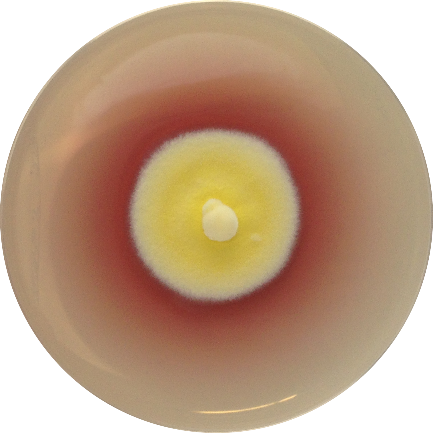


## Technical Details Light box

### Software Interface Light Box


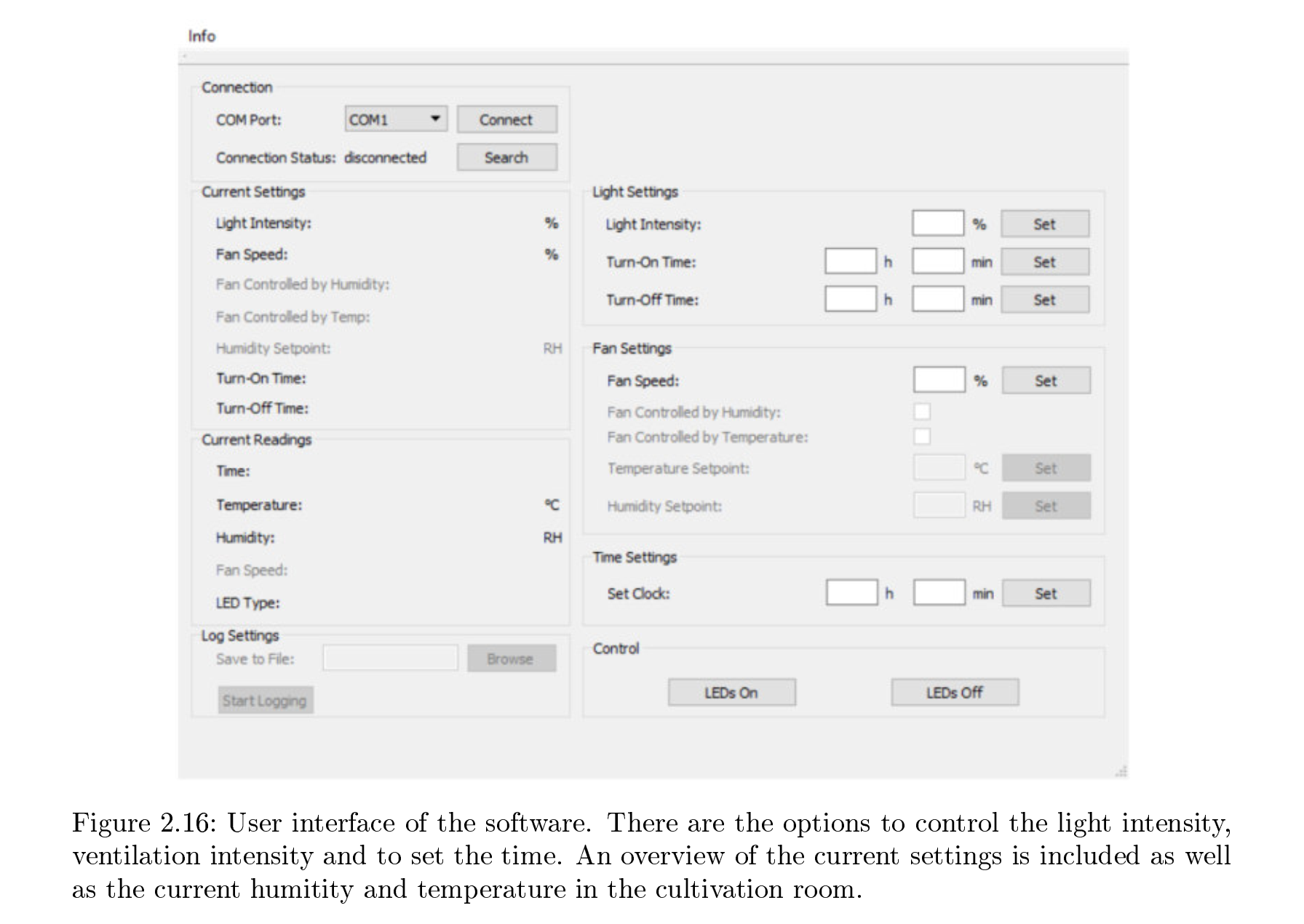


**Supplementary Figure S1:** User interface of the operation software with options to control the light intensity, the irradiation interval and the ventilation intensity. Besidean overview of the current settings also the current air humidity and the temperature of the cultivation chamber is provided.

### Illumination Properties Light Box System

**Supplementary Table S1** Relevant illumination properties of the used light sources. The LED wavelength and the respective FWHM were recorded as described in the section Materials and Method.

| Light source | Color | $\lambda_{peak}$ | $\Delta\lambda$  (FWHM) | Viewing angle |
| --- | --- | --- | --- | --- |
|  |  | nm | nm | ° |
| Cree XLamp XP-E2 SMD LED 2.6 V, 56.8 lm, 3000 mW | red | 635 | 18 | 130 |
| Würth WL-SMDC SMD High-Power LED 3.4 V, 85 lm | green | 519 | 38.1 | 125 |
| Cree XLamp XT-E SMD High-Power LED 2.4 V, 5000 mW | blue | 452 | 18.7 | 140 |


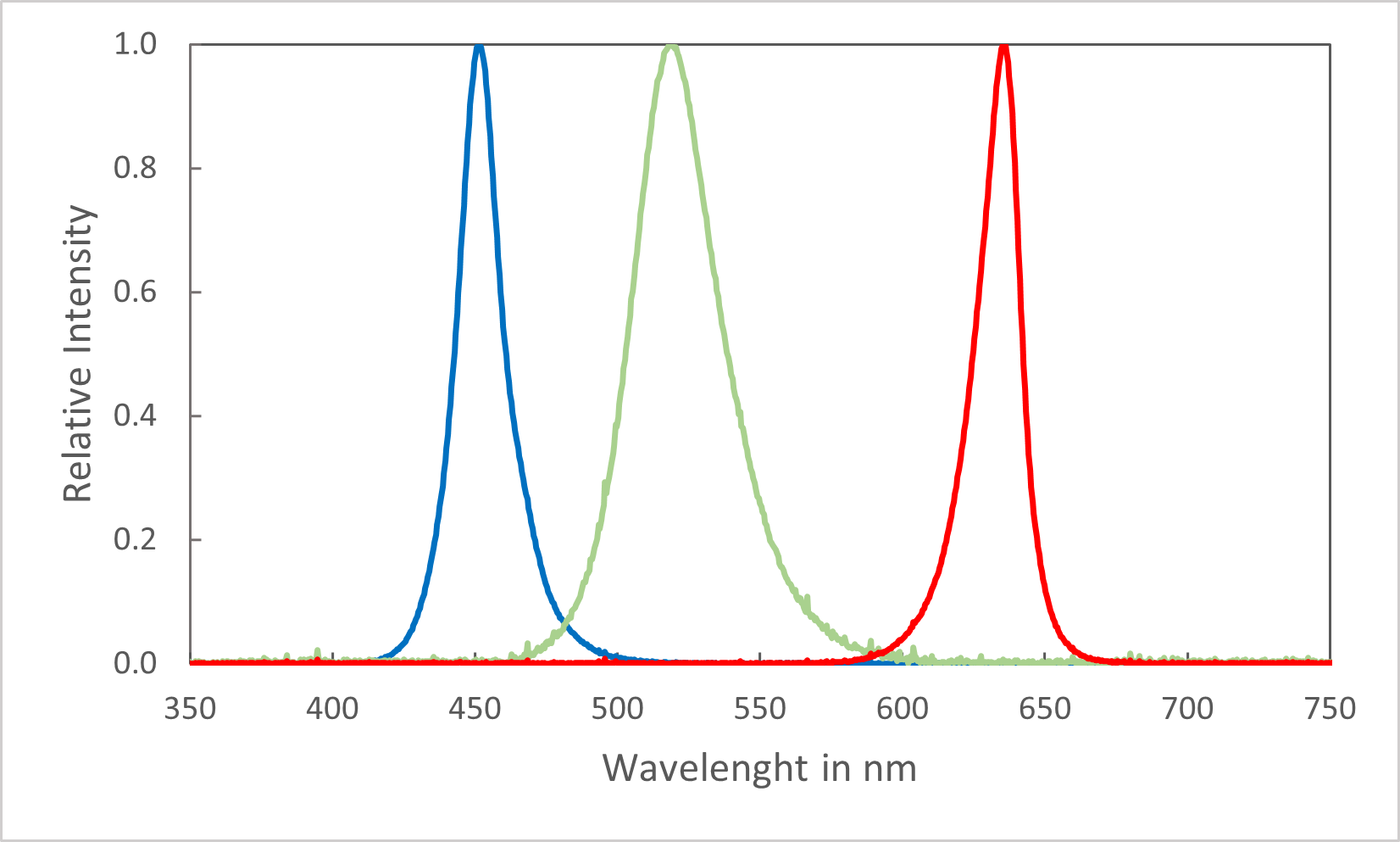


**Supplementary Figure S2:** Spectral distribution of the different irradiation scenarios used in this work. For better comparison, intensities were normalized.

### Calibration Intensity

Supplementary Figure S3: Calibration curve software setting of the light box versus measured intensity. Data are means of triplicate measurements.

### Illumination Homogeneity Petri-Dish Plates


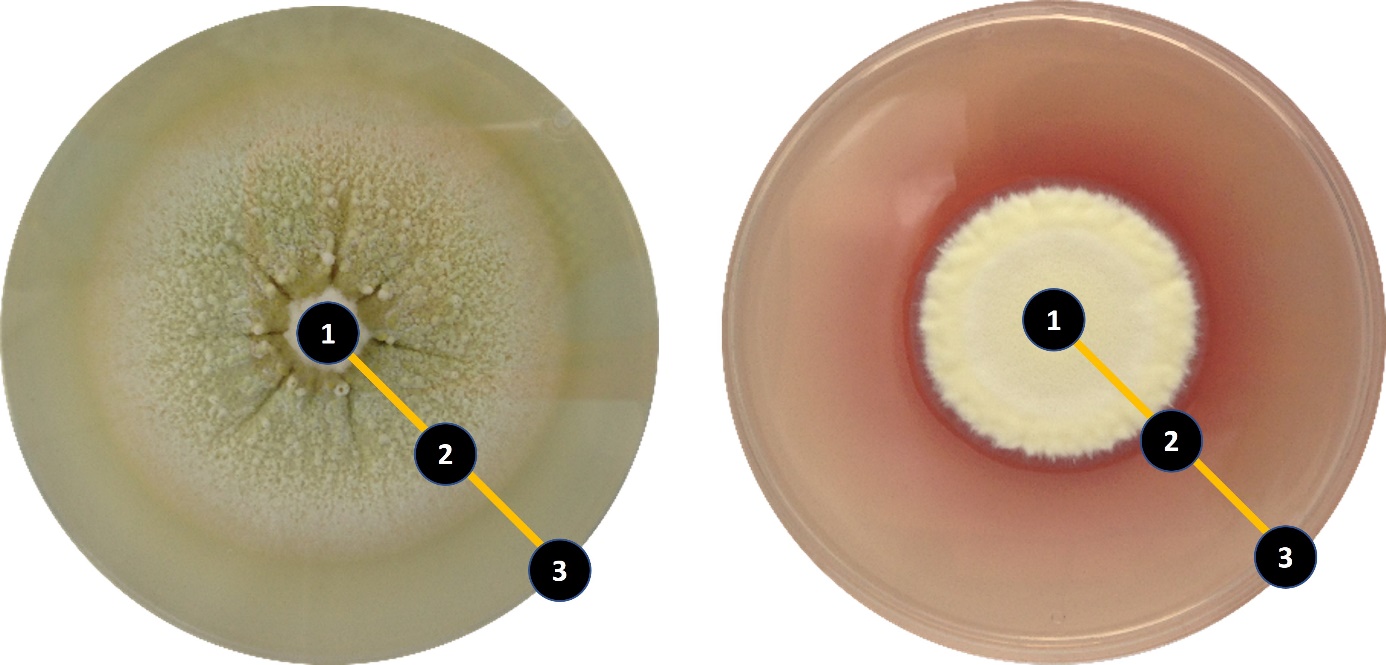


Supplementary Figure S4: Measuring points within one Petri-dish position to evaluate the light distribution within a Petri-dish. (1) center, (2) half radius, (3) border measuring point. Typical *M. brunneum* (left) and *B. brongniartii* (right) cultures grown at 25 °C for two weeks.

**Supplementary Table S2**: Light distribution measured at the center, the half-radius and the border of a Petri dish. The illumination was set to 30% for all tested colors. Data are means of triplicate measurements.

| Color | $\lambda_{peak}$ |  |  | Measuring points |  |
| --- | --- | --- | --- | --- | --- |
|  | nm |  | center  µW cm^-2^ | half radius  µW cm^-2^ | border  mW cm^-2^ |
| red | 635 |  | 151.0 ± 1.1 | 143.6 ± 0.3 | 76.0 ± 2.1 |
| green | 519 |  | 99.3 ± 0.3 | 93.9 ± 0.3 | 24.7 ± 0.3 |
| blue | 452 |  | 179.7 ± 0.1 | 168.7 ± 0.4 | 89.8 ± 2.6 |
|  |  |  |  |  |  |

## DAtA Experiments

**Supplementary Table S3:** Radial growth and conidia production of *M. brunneum* after two-week incubation on S4G at 25 °C under different light regimes. Data are means of triplicates.

| Light regime | Setting light box | Intensity | Mean diameter | Mean conidia |
| --- | --- | --- | --- | --- |
|  | % | µW cm^-2^ | mm | conida cm^-2^ |
| dark | - | - | 61.8 ± 0.9 | 0.04 * 10^6^ ± 0.030 * 10^6^ |
|  | 5 | 22.1 ± 0.1 | 63.6 ± 0.5 | 0.04 * 10^6^ ± 0.015 * 10^6^ |
| red (635 nm) | 10 | 66.9 ± 0.2 | 64.3 ± 0.6 | 0.24 * 10^6^ ± 0.150 * 10^6^ |
|  | 30 | 136.5 ± 0.3 | 62.3 ± 0.5 | 0.42 * 10^6^ ± 0.075 * 10^6^ |
|  | 5 | 16.5 ± 0.1 | 69.0 ± 0.4 | 2.20 * 10^7^ ± 1.200 * 10^7^ |
| green (519 nm) | 10 | 23.1 ± 0.0 | 69.2 ± 0.6 | 4.30 * 10^7^ ± 2.000 * 10^7^ |
|  | 30 | 96.2 ± 0.1 | 70.9 ± 0.2 | 0.55 * 10^7^ ± 0.251 * 10^7^ |
|  | 5 | 56.1 ± 0.2 | 66.8 ± 0.4 | 2.50 * 10^7^ ± 0.391 * 10^7^ |
| blue (452 nm) | 10 | 93.3 ± 0.4 | 67.9 ± 0.6 | 0.73 * 10^7^ ± 0.229 * 10^7^ |
|  | 30 | 188.9 ± 0.6 | 65.0 ± 1.6 | 0.09 * 10^7^ ± 0.033 * 10^7^ |

**Supplementary Table S4:** Radial growth and conidia production of *B. brongniartii* after two-week incubation on S2G at 25 °C under different light regimes. Data are means of triplicates.

| Light regime | Setting light box | Intensity | Mean diameter | Mean conidia |
| --- | --- | --- | --- | --- |
|  | % | µW cm^-2^ | mm | conida cm^-2^ |
| dark | - | - | 39.3 ± 0.6 | 1.70 * 10^7^ ± 0.459 * 10^7^ |
|  | 5 | 22.1 ± 0.1 | 37.0 ± 0.7 | 1.40 * 10^7^ ± 0.342 * 10^7^ |
| red (635 nm) | 10 | 66.9 ± 0.2 | 37.7 ± 2.1 | 1.80 * 10^7^ ± 0.661 * 10^7^ |
|  | 30 | 136.5 ± 0.3 | 36.1 ± 2.1 | 0.93 * 10^7^ ± 0.356 * 10^7^ |
|  | 5 | 16.5 ± 0.1 | 38.5 ± 0.6 | 8.70 * 10^7^ ± 1.700 * 10^7^ |
| green (519 nm) | 10 | 23.1 ± 0.0 | 38.1 ± 1.2 | 5.90 * 10^7^ ± 0.350 * 10^7^ |
|  | 30 | 96.2 ± 0.1 | 36.8 ± 1.0 | 1.10 * 10^7^ ± 0.368 * 10^7^ |
|  | 5 | 56.1 ± 0.2 | 39.3 ± 1.7 | 0.22 * 10^7^ ± 0.096 * 10^7^ |
| blue (452 nm) | 10 | 93.3 ± 0.4 | 37.2 ± 1.8 | 0.28 * 10^7^ ± 0.240 * 10^7^ |
|  | 30 | 188.9 ± 0.6 | 28.5 ± 5.1 | 0.14 * 10^7^ ± 0.023 * 10^7^ |

**Supplementary Table S5**: Pearson correlation coefficients (r) for the relation of diameter and conidia per cm^-2^ with the intensity of different light regimes.

| Light regime | *M. brunneum* | | | | *B. brongniartii* | | | |
| --- | --- | --- | --- | --- | --- | --- | --- | --- |
|  | diameter | | conidia cm^-2^ | | diameter | | conidia cm^-2^ | |
|  | r | p | r | p | r | p | r | p |
| Red | -0.76 | 0.004 | 0.82 | 0.001 | -0.30 | 0.269 | -0.48 | 0.070 |
| Green | 0.92 | <0.001 | -0.57 | 0.028 | -0.68 | 0.015 | -0.83 | <0.001 |
| Blue | -0.67 | 0.009 | -0.83 | <0.001 | -0.85 | <0.001 | -0.31 | 0.305 |

## Analytics

**
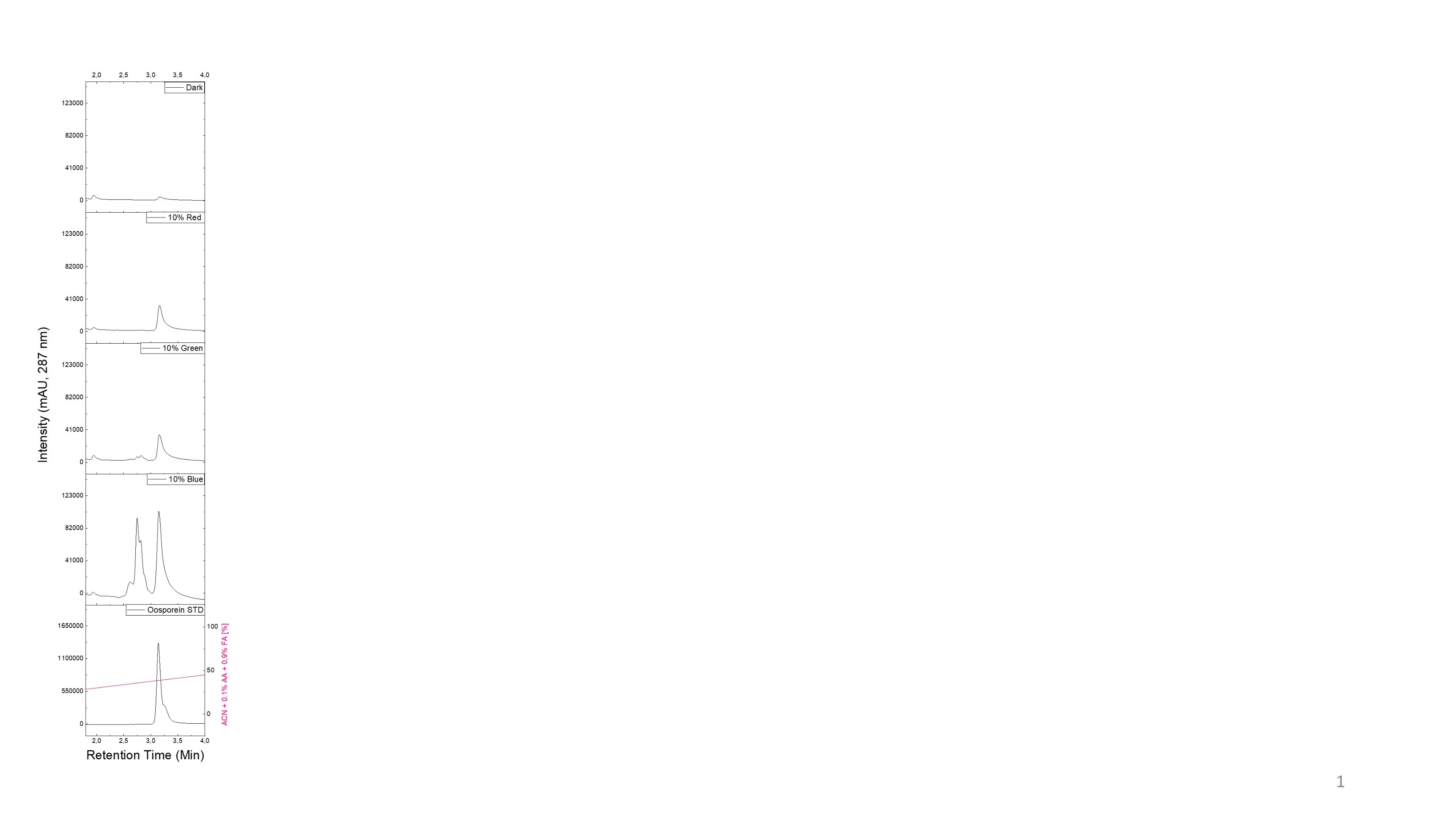
**

Supplementary Figure S5: Representative HPLC-DAD chromatograms (λ_det_ = 287 nm) of the targeted oosporein analysis of *B. brongniartii*. Stationary phase: Phenomenex Synergi 4u Hydro-RP 80A 150 x 4.6 mm. Mobile phase: Water (A) and ACN (B) supplemented with 0.1% acidic acid (AA) and 0.9% formic acid (FA).


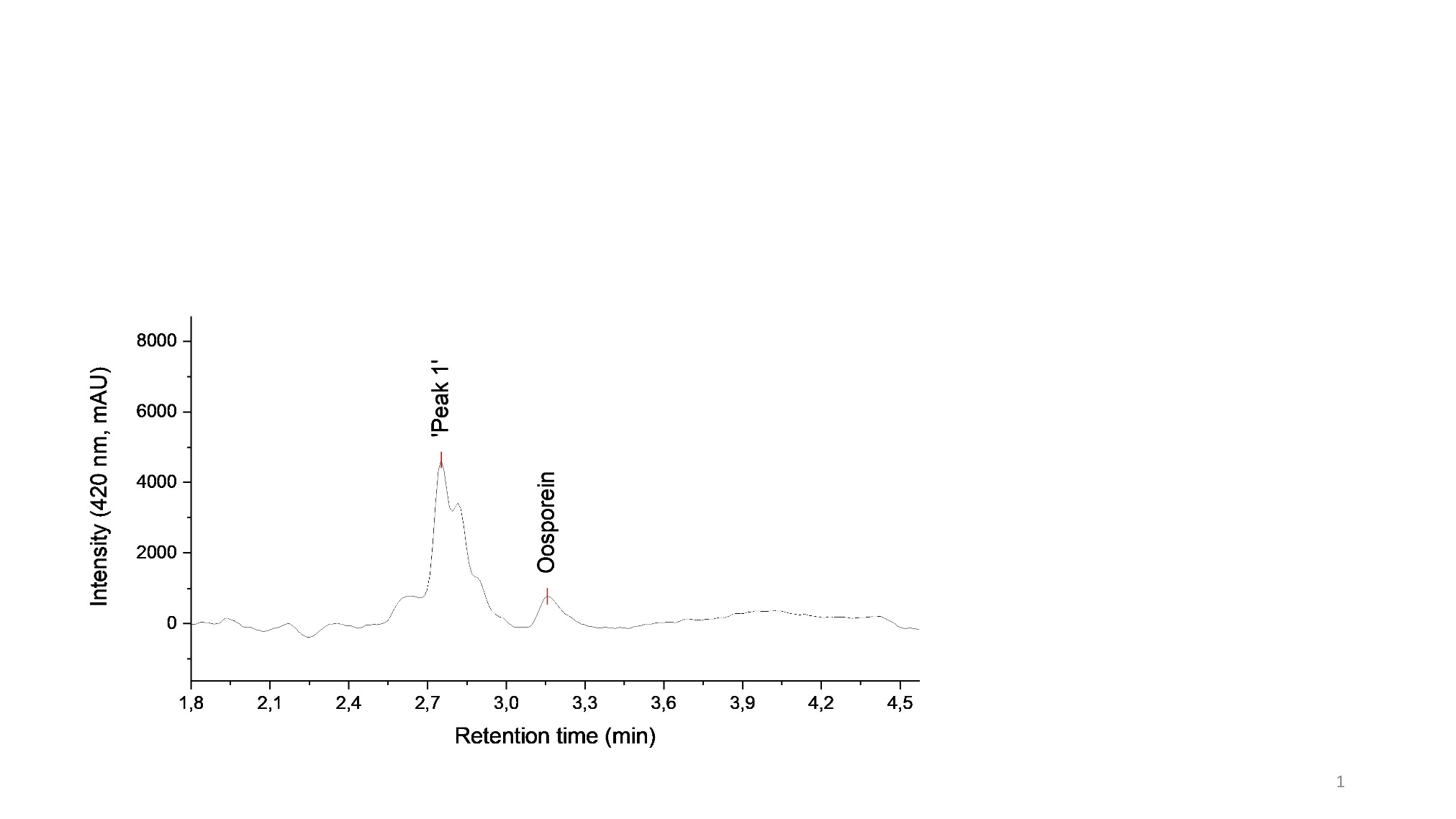


Supplementary Figure S6: HPLC-DAD chromatograms (λ_det_ = 420 nm) of the analyzed *B. brongniartii* extract grown under blue light (10%). A blue light-absorbing peak was detected at 2.77 min. The peak shape indicates a mixture of different compounds or isomeric forms. Stationary phase: Phenomenex Synergi 4u Hydro-RP 80A 150 x 4.6 mm. Mobile phase: Water (A) and ACN (B) supplemented with 0.1% acidic acid (AA) and 0.9% formic acid (FA).
